# Essential Oils in Foods: From Ancient Times to the 21st Century

**DOI:** 10.3390/foods5020043

**Published:** 2016-06-14

**Authors:** Esther Sendra

**Affiliations:** IPOA Research Group (UMH-1 and REVIV-Generalitat Valenciana), AgroFood Technology Department, Escuela Politécnica Superior de Orihuela, Miguel Hernández University, Ctra. Beniel km. 3,2. E-03312 Orihuela, Alicante, Spain; esther.sendra@umh.es; Tel.: +34-966-749-735; Fax: +34-966-749-677

Medicinal plants and culinary herbs have been used since ancient times. Essential oils (EO) are a mixture of numerous compounds, mainly terpenes, alcohols, acids, esters, epoxides, aldehydes, ketones, amines and sulfides, that are probably produced by plants as a response to stress [1]. Their use is linked to cultural heritage and there is a huge variety of plants and uses. In the last 20 years, probably linked with advances in chemical analysis (especially chromatography and detection systems), many studies have been undertaken to investigate the composition of essential oils (EOs) and plant extracts. Different countries and territories have different endemic plants or varieties that may provide a huge spectrum of components in completely different amounts and proportions and that have reported food, cosmetic and medicinal uses [2,3,4,5,6]. The comprehensive knowledge of worldwide herbs and plants of culinary and medicinal interest may benefit consumers by providing natural preserving agents, as well as territories, given that it may add value or increase demand for specific plants endemic of such territories and so promote rural development.

EOs have been used in foods as flavoring, as well as preserving agents, due to antimicrobial and antioxidant properties. Their main active components are: thymol, carvacrol, eugenol, cinnamaldehyde and linalool [7], although their mechanism of action is still poorly understood [1,8]. Microbial safety of foods is of major concern for consumers, regulatory agencies and food industries. Food contamination and spoilage have not yet been solved and the contribution of EOs is highly valuable in a scenario of increasing demand of minimally processed foods and natural preservatives. The main limitations of EO uses in foods are: causing sensory changes in foods (due to their strong odor and flavor and their color, which may taint foods), EOs from the same plant species presenting a high variability in quality and quantity of bioactive constituents, and such bioactive compounds potentially being lost or reduced by many food processing techniques or even EO extraction techniques [7,9]. In contrast, the main advantages of EO incorporation into foods are: EOs are natural compounds that, due to their antioxidant and antimicrobial effects, may reduce the use of synthetic preservatives, they are suitable for organic foods, they allow the use of clean labels on foods, they are in line with the trend towards green consumerism, they are generally recognized as safe and have a long history of being consumed by humans [7].

Studies on the biological activities of essential oils have become increasingly important in the search for natural and safe alternative preservatives and health promoters. Another relevant use of EOs is their incorporation as feed supplements: for ruminants to modify ruminal metabolism in order to mitigate methane and ammonia emissions [10] and for monogastrics due to their promising function as alternatives for replacing antibiotic growth promoters given their positive impact on growth performance as well as gut microbiota [11]. Their mode of action in animal growth is poorly understood and still needs deeper investigation [9,10,11].

There is an abundance of scientific literature on the study of *in vitro* antioxidant and antimicrobial properties of EOs [2,3,4,5,6,12,13], a lot of research is now being undertaken on studies on food [14,15] and the interaction between food matrices and EOs that may affect their activity [1]. Recent developments in the use of EOs in foods point to their inclusion in systems of multiple hurdle technologies to assure food safety. Some examples are the development of edible films with EOs, encapsulation of EOs, the incorporation of the EO in active packages [7,16], or their combination with other natural compounds to enhance the preservation function while reducing sensory changes [17]. Actual and future research in EOs for food preservation will focus upon the search for active compounds combinations to reduce sensory impact while assuring effectiveness, active packaging technologies (engineering aspects of EO inclusion in multilayer and complex packages), expanding the knowledge of plants endemic to different ecosystems, exploring new uses in foods (like nutraceuticals) or new active compounds (after assuring regulatory and safety issues), and studying the impact of EOs introduction on the microbial ecology of foods, among others. Definitively, EOs have a long history of being used in foods and they still have a relevant place in the present and future of food science and technology.
